# Exome capture sequencing identifies a novel mutation in *BBS4*

**Published:** 2011-12-30

**Authors:** Hui Wang, Xianfeng Chen, Lynn Dudinsky, Claire Patenia, Yiyun Chen, Yumei Li, Yue Wei, Emad B. Abboud, Ali A. Al-Rajhi, Richard Alan Lewis, James R. Lupski, Graeme Mardon, Richard A. Gibbs, Brian D. Perkins, Rui Chen

**Affiliations:** 1 Human Genome Sequencing Center, Baylor College of Medicine, Houston, TX; 2 Department of Molecular and Human Genetics, Baylor College of Medicine, Houston, TX; 3 Department of Ophthalmology, Baylor College of Medicine, Houston, TX; 4 Texas Children’s Hospital, Baylor College of Medicine, Houston, TX; 5 Department of Neurology, Baylor College of Medicine, Houston, TX; 6 Department of Neuroscience, Baylor College of Medicine, Houston, TX; 7 Department of Pathology, Baylor College of Medicine, Houston, TX; 8 Program in Developmental Biology, Baylor College of Medicine, Houston, TX; 9 Department of Biology, Texas A&M University, College Station, TX; 10 Leukemia Department, University of Texas, M. D. Anderson Cancer Center, Houston, TX; 11 King Khaled Eye Specialist Hospital, Riyadh, Kingdom of Saudi Arabia

## Abstract

**Purpose:**

Leber congenital amaurosis (LCA) is one of the most severe eye dystrophies characterized by severe vision loss at an early stage and accounts for approximately 5% of all retinal dystrophies. The purpose of this study was to identify a novel LCA disease allele or gene and to develop an approach combining genetic mapping with whole exome sequencing.

**Methods:**

Three patients from King Khaled Eye Specialist Hospital (KKESH205) underwent whole genome single nucleotide polymorphism genotyping, and a single candidate region was identified. Taking advantage of next-generation high-throughput DNA sequencing technologies, whole exome capture sequencing was performed on patient KKESH205#7. Sanger direct sequencing was used during the validation step. The zebrafish model was used to examine the function of the mutant allele.

**Results:**

A novel missense mutation in Bardet-Biedl syndrome 4 protein (*BBS4*) was identified in a consanguineous family from Saudi Arabia. This missense mutation in the fifth exon (c.253G>C;p.E85Q) of *BBS4* is likely a disease-causing mutation as it segregates with the disease. The mutation is not found in the single nucleotide polymorphism (SNP) database, the 1000 Genomes Project, or matching normal controls. Functional analysis of this mutation in zebrafish indicates that the G253C allele is pathogenic. Coinjection of the G253C allele cannot rescue the mislocalization of rhodopsin in the retina when *BBS4* is knocked down by morpholino injection. Immunofluorescence analysis in cell culture shows that this missense mutation in *BBS4* does not cause obvious defects in protein expression or pericentriolar localization.

**Conclusions:**

This mutation likely mainly reduces or abolishes *BBS4* function in the retina. Further studies of this allele will provide important insights concerning the pleiotropic nature of *BBS4* function.

## Introduction

The molecular mechanisms underlying genetic diseases are often highly heterogeneous. Specifically, mutations in different genes can result in the same clinical phenotype. In addition, patients with different mutations, or even the same mutation, in one gene can manifest different clinical phenotypes. This underscores the importance of comprehensive documentation of phenotype and genotype relationships as the first step in accurate and personalized treatment of the disease. Leber congenital amaurosis (LCA; OMIM 204000) most often presents as a recessive disease, is one of the most severe forms of vision loss, is apparent by 1 year of age, and accounts for more than 5% of all retinal dystrophies [1,2]. The clinical features of this genetically heterogeneous disease include severe and early visual loss, sensory nystagmus, amaurotic pupils, and absent electrical signals on electroretinogram (ERG) [1,3]. To date, at least 15 genes have been associated with LCA, and they act in strikingly diverse genetic and functional pathways, including retina development, phototransduction, vitamin A metabolism, protein transport, and centrosome formation [4-11]. As a result, accurate molecular diagnosis of the disease is essential for developing and providing proper treatment to the patient. Indeed, gene and drug therapy have recently been developed specifically for patients with mutations in retinal pigment epithelium 65 (*RPE65*) [12,13].

Despite the substantial efforts in human mapping studies during the last decade, about 35% of LCA familial cases in the European population cannot be accounted for by mutations in the 15 known LCA genes. The portion of LCA patients with unknown mutations in other populations may be significantly higher [7,14]. These cases can be partially explained by novel LCA disease genes. Gene identification becomes increasingly difficult as each likely accounts for only a small fraction of the patients with this rare disease. Some of these patients may carry mutations in other known retinal disease genes not been previously linked to LCA. This is particularly likely for genes normally associated with syndromic retinal degenerative diseases. Indeed, researchers have reported that several syndromic diseases, such as Alström syndrome, Batten disease, Joubert syndrome (JBTS), peroxisomal diseases, and Senior-Loken syndrome (SLSN), show an “LCA-like ocular phenotype” [6]. The mutations in some known LCA disease genes can also cause syndromic diseases. For example, Centrosomal protein of 290 kDa (*CEP290*, also known as *NPHP6*), which represents the most common cause of LCA identified to date [15,16], is also associated with other diseases, such as retinitis pigmentosa [17], Meckel syndrome (MKS) [18,19], SLSN [20], Bardet-Biedl syndrome (BBS) [21] and JBTS [22,23]. Similarly, Bardet–Biedl syndrome protein-8 (*BBS8*), one of the genes involved in BBS, has been linked to retinitis pigmentosa [24].

Since more than 165 genes have been associated with retinal diseases (RetNet), screening for mutations in all of these genes with traditional Sanger sequencing is cost prohibitive. The recent development of next-generation sequencing technology provides a much faster and more cost-effective alternate approach for identifying causative mutations [25-28]. For example, the entire exome of an individual can be sequenced at great depth with a single lane of the Illumina HiSeq 2000 sequencer coupled with DNA capture technology [29-32]. Indeed, several disease-causing mutations have been identified with exome sequencing of a small number of patients, underscoring the great potential of this technology [33-41].

To test the feasibility of identifying disease-causing mutations with direct sequencing, we employed a combination of genetic mapping and the whole exome sequencing approach. Based on single nucleotide polymorphism (SNP) mapping, we first mapped the disease locus of a Saudi family to an 11.2 Mb region on chromosome 15. With whole exome capture and sequencing, we identified a missense mutation in *BBS4* (NM_033028), one of the 14 genes known to cause BBS (OMIM 209900) [42]. This mutation was further confirmed with Sanger sequencing and family segregation. Bbs4^-^ zebrafish was used to determine the functional relevance of this allele. In contrast, the mutant *BBS4* mRNA with the missense allele cannot rescue the gastrulation and the retinal phenotype, while the wild-type human *BBS4* mRNA can successfully rescue the gastrulation and the retinal phenotype. Our results are the first report that links *BBS4* with LCA and indicates that partial loss of *BBS4* function can cause an LCA-like phenotype with quite mild syndromic features.

## Methods

### Subjects

The study was approved by the Human Subjects Ethics Subcommittee of Baylor College of Medicine. We obtained blood samples and pedigrees after receiving informed consent from all individuals. Approval was obtained from the institutional review boards of the participating centers. Family KKESH205 was recruited by Dr. Lewis through the King Khaled Eye Specialist Hospital (KKESH) in Riyadh, Saudi Arabia. Blood samples from all available family members were collected and processed at KKESH hospital in Riyadh, Saudi Arabia with the Qiagen (Germantown, MD) blood genomic DNA extraction kit following the protocol provided by the manufacturer.

### Whole exome capture and library construction

Five μg of genomic DNA from KKESH205#7 was used to make a SOLiD fragment library. After the SOLiD adaptors were ligated to the genomic DNA, five cycles of PCR amplification were applied. This DNA was then hybridized to oligonucleotides from NimbleGen’s (Madison, WI) liquid capture kit (the Consensus CDS design design) for 72 h to enrich exonic regions. Hybridized fragments were eluted and sequenced with single end 50 bp reads on a SOLiD platform.

### Sanger sequencing

Primers surrounding the variant were designed with the online program Primer3, and PCR products were purified with ExoSAP-IT (USB Corp, Cleveland, OH). Sequencing chemistry was performed using an ABI (Life Technology, Carlsbad, CA) PRISM Big Dye Terminator Cycle Sequencing Ready Reaction Kit (v3.1), the PCR amplicon sequenced on an ABI 3700, and the results analyzed using Sequencher software.

### Morpholino knockdown

Wild-type zebrafish of an AB/Ekkwill hybrid strain were housed, bred, and staged according to standard procedures [43]. Wild-type embryos (n=280–300) were injected at the 1–2 cell stage with 4.0 ng of a *bbs4* morpholino (GeneTools, Philomath, OR) designed to block translation of *bbs4* mRNA (5′-GAA AAA GAT CAC TAC TGT AAA GCA T-3′). To quantify our results, we used a classification scheme similar to one previously described [44]. Embryos with normal somites and notochord but a shortened body axis were considered mild, whereas embryos with broad, flattened somites and severely kinked notochords were considered severe.

### Immunohistochemistry

Larvae were fixed in 4% paraformaldehyde in PBS (137 mM NaCl, 10 mM phosphate, 2.7 mM KCl, pH 7.4) for 2–4 h at 4 °C. Following fixation, samples were washed in PBST (PBS + 0.01% Tween-20) and equilibrated with 30% sucrose/PBS at 4 °C. Samples were oriented in 100% Tissue-Tek OCT (Miles, Inc., Elkhart, IN) and frozen at −20 °C. Cryosections (10 μm thick) were mounted on gelatin-coated slides, and dried for 2–3 h at room temperature. Slide edges were lined with a hydrophobic marker (PAP pen) and washed with PBS before blocking for 1 h (PBS + 0.05% Tween-20 + 0.1% dimethyl sulfoxide + 0.1% BSA). The 1D1 antibody (gift of Dr. James Fadool, Florida State University, Tallahassee, FL) was diluted 1:100 in blocking solution and incubated overnight at 4 °C in a humid chamber. Slides were washed with PBST before incubation with the appropriate fluorescent-conjugated secondary antibodies. Slides were counterstained with 4',6-diamidino-2-phenylindole (DAPI; Invitrogen, Carlsbad, CA) to label DNA. Slides were washed 3× for 10 min in PBST, and coverslips were mounted with Vectashield (Invitrogen). Images were obtained using a Zeiss AxioImager (Zeiss, Thornwood, NY) compound microscope with an ApoTome attached.

### RNA synthesis

The wild-type and mutant *BBS4* alleles were amplified with PCR and cloned into pBluescript (Fermentas, Inc., Glen Burnie, MD). The plasmids were linearized with BamH1, purified, and used as a template for in vitro transcription to generate 5′-capped mRNAs with the mMESSAGE mMACHINE kit (Ambion, Austin, TX). Synthetic mRNA (150–300 pg) was injected alone or coinjected with morpholinos.

### In situ hybridization

Digoxigenin-labeled antisense riboprobes were synthesized from full-length cDNAs using standard protocols. The *pax2.1* and *myoD* full length cDNAs were gifts from Dr. Arne Lekven (Texas A&M University, College Station, TX). In situ hybridizations were performed as previously described [45].

### Cell transfection

HeLa cells were transfected with 1 µg of *pCMV-BBS4* cDNA and used for immunostaining 24 h later. HeLa cells were fixed with methanol/acetone (1:1) at room temperature for 10 min and blocked with PBS-10% fetal bovine serum (FBS) for 10 min before primary antibody was added. Primary antibodies (mouse anti-FLAG 1:100 and rabbit antigamma-tubulin, Sigma, St. Louis, MO) in PBS-5% FBS were incubated with the cells at 37 °C for 2 h, followed by washing. The cells were incubated with secondary antibodies (Annexin V-antimouse, Cy3-antirabbit) in PBS-5% FBS (1:1,000) at 37 °C for 1 h. The cells were washed, DAPI stained, and mounted.

### Bioinformatic analyses

To analyze the large amount of exome sequencing data, a semiautomated pipeline was constructed to identify putative disease-causing mutations. First, all reads were mapped to the human reference genome (NCBI build 36) using BWA (Ver. 0.5.0) with default parameters [46]. Reads that mapped to multiple positions in the genome were excluded from further analysis. Second, putative variants, including single nucleotide variants or insertions or deletions (in/dels), were identified using Samtools [47]. A cutoff score of 40 or variance bases at both strands with a confidence score greater than 30 were used to exclude low-quality variants. Furthermore, only variants with allele frequencies greater than 15% were kept.

## Results

### Clinical features

**Figure 1:**
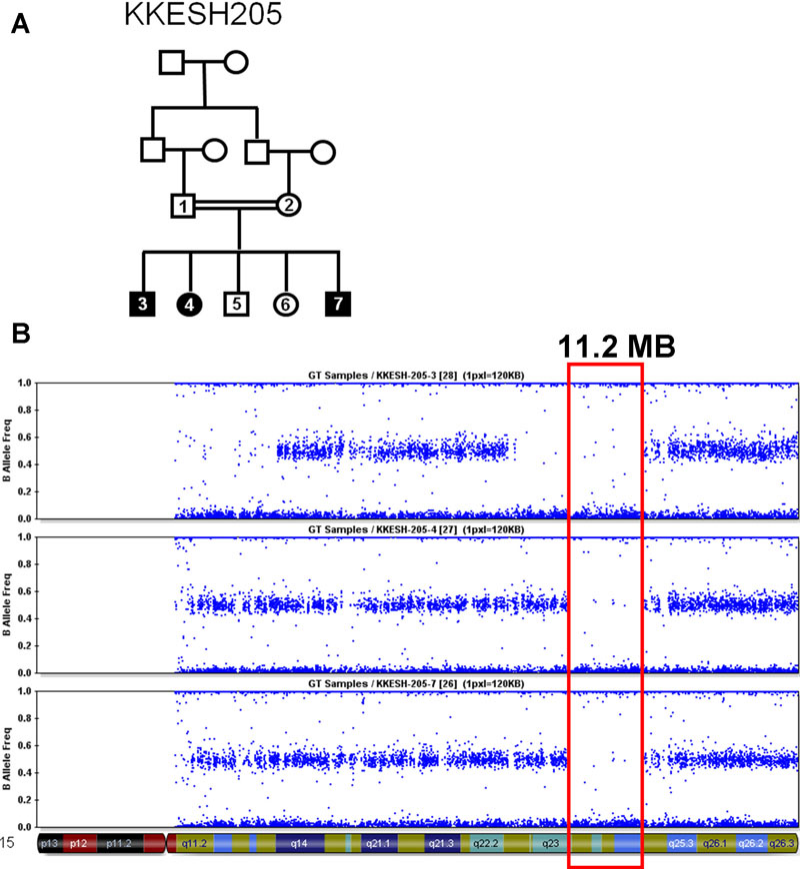
Pedigree and homozygosity mapping of KKESH205. **A**: Pedigree of the KKESH205 family with LCA is shown. Affected, solid symbols; unaffected, open symbols; squares, male; circles, female. **B**: Homozygosity mapping of KKESH205 using high-density SNP arrays. The 11.2 Mb region, which is located on chromosome 15 (red box), is the only homozygous region shared by all three affected members in the whole genome.

KKESH205 is a consanguineous Saudi Arabian family. Within the family, there are three affected and four unaffected members (Figure 1A). All affected members of KKESH205 show typical LCA phenotypes, including lack of ERG, infantile nystagmus, and pigmentary retinopathy with a “rubelliform” appearance. In addition, several mild phenotypes have been observed, including delayed walking (after age 2), slowness in learning speech (after age 3 and 4), and mild facial dysmorphism with bilateral enophthalmos.

### Homozygosity mapping

To identify the causative mutation in this family, we first performed direct Sanger sequencing to check all the coding exons from all 15 known LCA disease genes in one affected member of KKESH205. No homozygous mutations were identified within the coding regions of these 15 genes (data not shown). Therefore, homozygosity mapping of the disease locus was performed by genotyping all three affected members of KKESH205 using the Illumina 370K SNP array. A single 11.2 Mb homozygous region on chromosome 15 is shared by all three affected members, and therefore it almost certainly contains the disease locus (Figure 1B). Consistent with the Sanger sequencing described above, this region contains no known LCA disease genes. Due to the high gene density in this region, direct Sanger sequencing of all coding exons in this 11.2 Mb span was prohibitively expensive and labor intensive.

### Mutation detection

**Figure 2:**
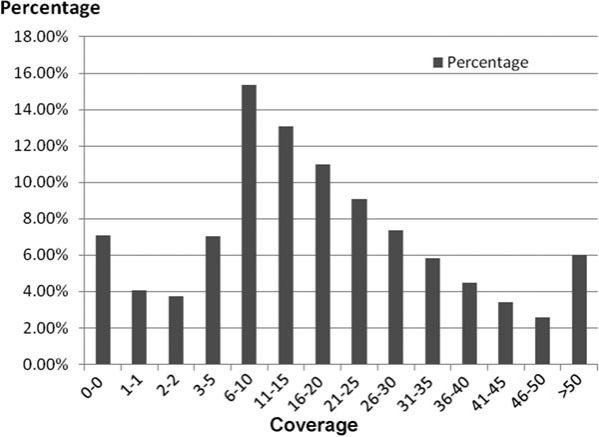
Distribution of sequencing coverage for the targeted region. A total of about 1.6 million reads uniquely map to the exons. Approximately 93% of the coding exons from the targeted region have at least 1X coverage.

Since the development of a custom designed regional capture is time-consuming and costly, we decided to apply whole-exome capture sequencing to one affected individual (KKESH205#7) while focusing the analysis on the candidate region to identify the disease-causing mutation in this family. A total of about 1.6 million reads uniquely map to the exons with an average of 18.6X coverage. Moreover, 93% of targeted exons have at least 1X coverage (Figure 2). Based on the filtering criteria (see Methods), a total of 370,000 SNPs and in/dels were identified for further analysis. Since disease-causing variants for LCA should be rare in the human population, common variants were filtered out. Variants found in the Single Nucleotide Polymorphism database (dbSNP database) or the 1000 Genomes SNP database at frequency greater than 0.5% were excluded, and the remaining variants are likely to be rare. As expected, most variants (96.4%) identified in this individual are indeed common. Finally, variants that did not lead to coding changes (i.e., synonymous) were excluded. Based on the current reference gene annotation, only variants that affect protein coding changes or splicing were subject to downstream analysis. As a result, 352 candidate variants, all of which are single nucleotide changes, were identified in the entire exome (Appendix 1).

**Figure 3:**
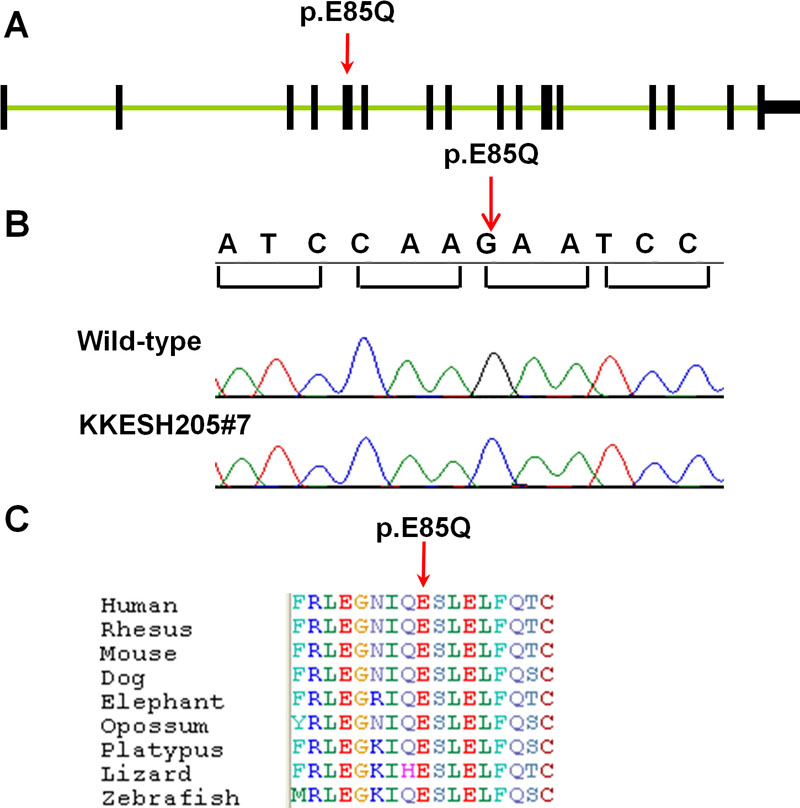
Gene structure of human *BBS4*. **A**: Exon-intron structure of *BBS4*. Exons are indicated as black boxes. The single missense mutation identified is located in the fifth exon (p.E85Q, red arrow). **B**: Sequence traces of wild-type and affected family members. A homozygous mutation from G to C was identified in affected member KKESH205#7 (red arrow). **C**: Amino acid alignment of a portion of the predicted BBS4 protein from nine different vertebrate species. The mutated amino acid is indicated (p.E85Q, red arrow).

As described previously, homozygous mapping of this consanguineous KKESH205 family via high-density SNP genotyping arrays identified a single critical region on chromosome 15. Inspection of the 352 candidate variants revealed only one homozygous missense change in this locus. This missense mutation (G-C) is located in the fifth exon of *BBS4*, a gene known to cause BBS, a rare human genetic disorder characterized by obesity, retinal dystrophy, renal anomalies, hypogenitalism, polydactyly, and numerous developmental and behavioral defects [48]. As ocular phenotypes are a common clinical feature of BBS and LCA, further analysis of this candidate mutation was performed. First, this mutation was confirmed with Sanger sequencing (Figure 3B). Second, segregation of this missense mutation in *BBS4* within this LCA family was examined. All members from KKESH205 were genotyped for this mutation with Sanger sequencing; as expected, this allele segregated precisely with the disorder (Figure 3B). Third, to further test if this mutation is indeed rare in the Saudi population, Sanger sequencing was performed on 200 normal matching controls, including 96 from Saudi Arabia. As expected, this mutation was not observed in the control population, indicating that the allele is rare. Furthermore, this mutation is not observed in the dbSNP130 and 1,000 Genomes database, indicating that the variant is very rare. This G->C mutation changes an amino acid (Glutamate to Glutamine) that is conserved from lizard to human (Figure 3C). A damage score of 4.91 was assigned to this position based on the SeattleSNPs. Since loss of *BBS4* function is known to cause BBS and *BBS4* knockout mice show severe retinal dystrophy, this novel missense mutation in *BBS4* is likely to be pathologic in the KKESH205 family [49].

### Functional study of the mutant allele

**Figure 4:**
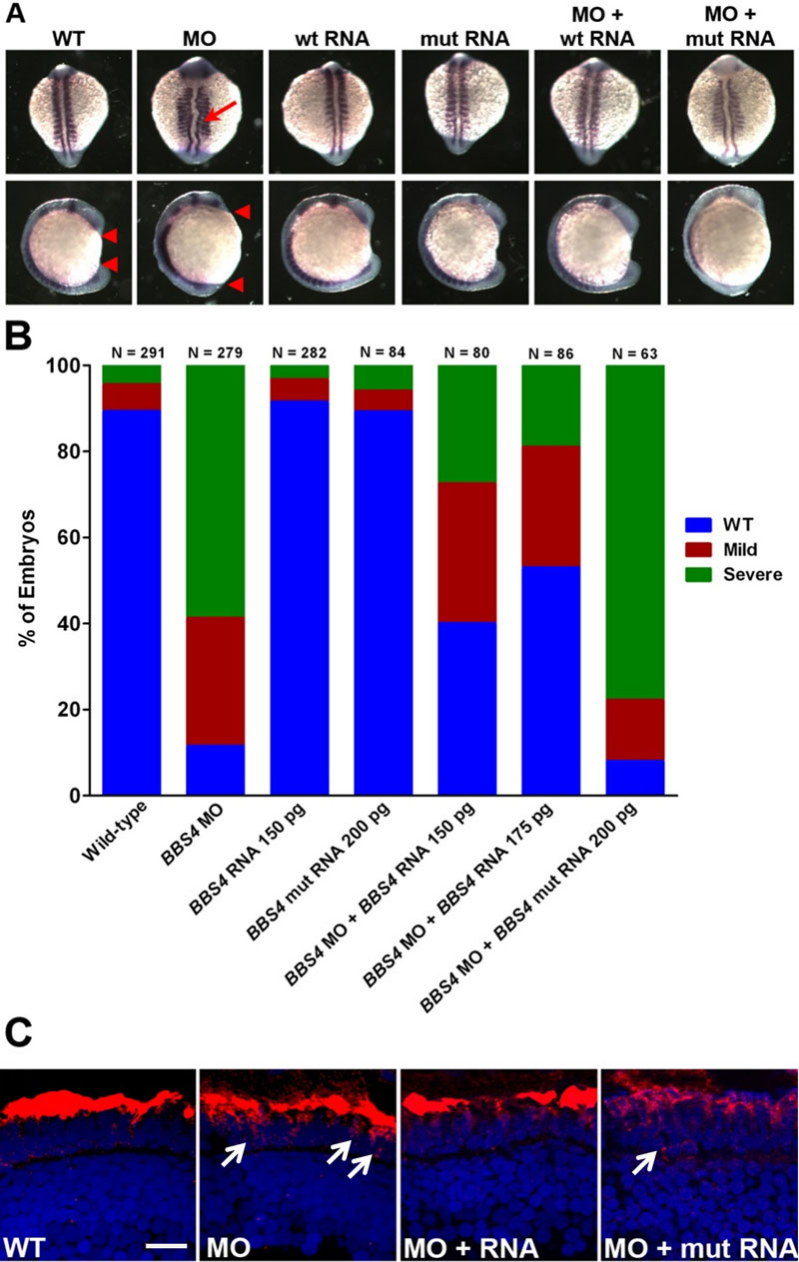
*BBS4* is required for normal zebrafish development and rhodopsin localization. **A**: Representative examples of whole-mounted wild-type (WT) embryos, morpholino-injected (MO) embryos, embryos injected with wild-type (wt) or mutant (mut) human *BBS4* mRNA, or embryos coinjected with morpholino and mRNA. Dorsal view (top row) and lateral view (bottom row) are shown of embryos at the 12–14 somite stage following in situ hybridization with *pax2a* and *myoD* riboprobes. Embryos were categorized phenotypically based on shortened body axis (anterior and posterior ends marked by red triangles) and notochord defects (red arrow). **B**: Quantification of the efficiency of rescue from gastrulation defects following coinjection of *BBS4* morpholino (MO) and mRNA. The number of animals analyzed for each group is noted above each bar. **C**: Retinal cryosections of 5 dpf zebrafish retinas stained for rhodopsin (red). White arrows indicate rhodopsin mislocalization (Scale bar=10 μm).

To further test if this missense mutation negatively affects *BBS4* gene function in vivo, we used zebrafish, a model system well established for assessing BBS gene function [42]. Suppression of cilia genes, including *BBS4*, in zebrafish by antisense morpholinos typically produce convergent-extension defects at the 12–14 cell stage, including a shortened body axis, undulating notochord, and broadened somites [44,50-53]. These phenotypes likely reflect abnormal planar cell polarity (PCP) signaling, a hypothesis supported by evidence that BBS proteins genetically and physically interact with components of the PCP pathway [52,54,55]. Researchers have proposed that defects in PCP signaling underlie some clinical features of BBS, including kidney cysts and hearing loss [50,54]. Wild-type embryos (n=280–300) were injected at the 1–2 cell stage with 4.0 ng of a *bbs4* morpholino previously shown to block translation of *bbs4* mRNA [50]. Consistent with previous results, *bbs4* morphants exhibited a shortened body axis (Figure 4A, red arrowheads), broadened somites, and a kinked notochord (Figure 4A, red arrow) at the 12–14 somite stage (approximately 15 h post-fertilization). These phenotypes were partially rescued by coinjection of wild-type human *BBS4* mRNA but not the *BBS4* missense allele. Overexpression of either the wild-type or mutant *BBS4* mRNA did not cause any phenotypes. Embryos with normal somites and notochord but a shortened body axis were considered mild, whereas embryos with broad, flattened somites and severely kinked notochords were considered severe. Of the morpholino-injected embryos, 59% had a severe phenotype, and 89% exhibited some defect (Figure 4B). Severe phenotypes were seen in only 27% of the embryos (n=80) when the *bbs4* morpholino was coinjected with 150 pg of wild-type human *BBS4* mRNA and in only 19% of the embryos (n=86) coinjected with 175 pg of wild-type *BBS4* mRNA. These results mirror previous reports of approximately 80% of morphants being rescued by human wild-type *BBS4* mRNA [50]. Importantly, injection of 200 pg of mRNA from the human *BBS4* missense allele did not provide any rescuing effect (n=63). These results indicate that the G253C allele is pathogenic and does not have any readily apparent activity in this assay.

The clinical phenotypes associated with this *BBS4* allele prompted us to investigate whether retinal phenotypes in zebrafish were associated with morpholino suppression of zebrafish *bbs4*. At 5 days post fertilization, zebrafish rod photoreceptors express high levels of rhodopsin that localizes almost exclusively to the photoreceptor outer segments (Figure 4C). To test if *bbs4* is involved in photoreceptors in zebrafish, retina from 15 embryos from each group as described above were sectioned and stained with rhodopsin. Injection of *bbs4* morpholinos resulted in partial mislocalization of rhodopsin to the inner segment. This result resembles the *Bbs4* knockout mouse [49,56], where photoreceptor outer segments were present but partial rhodopsin mislocalization occurred (Figure 4C, white arrows). When *bbs4* morphants were coinjected with 200 ng of human *BBS4* mRNA, the trafficking defect was reversed, and no rhodopsin mislocalization was observed. In contrast, rhodopsin remained mislocalized in embryos coinjected with morpholino and 200 ng of the *BBS4* missense mRNA.

**Figure 5:**
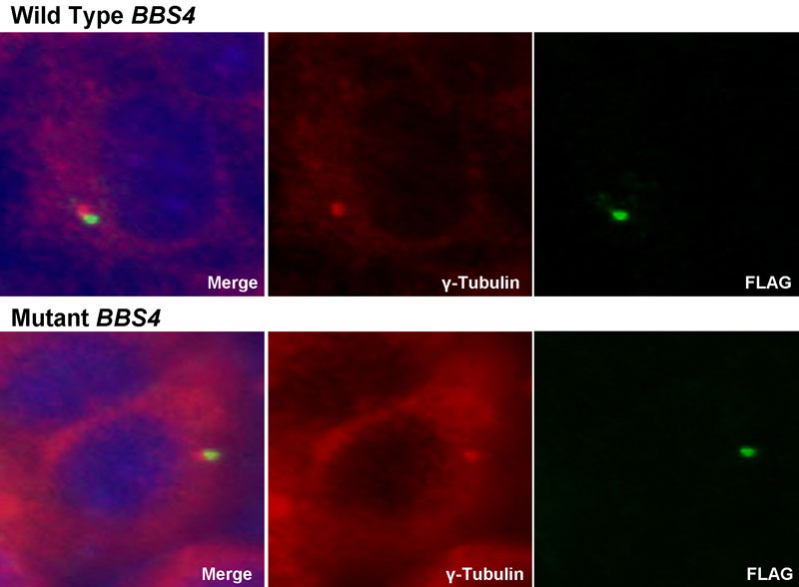
E235Q mutant protein is produced and correctly localizes to the pericentriolar region in HeLa cell culture. Immunofluorescence of FLAG-tagged wild-type and mutant BBS4 protein in HeLa cells. Cells were stained using an anti-FLAG antibody (green) for BBS4 expression and anti-γ tubulin antibody (red) for centrosomal localization. Nuclei were stained with DAPI.

One possible mechanism by which this missense mutation in *BBS4* could cause a functional defect is to affect protein folding, stability, or localization. To test this possibility, HeLa cells were transfected with wild-type or mutant full-length *BBS4* cDNA in the expression vector pCMV-Tag1 (Stratagene, La Jolla, CA). BBS4 normally localizes to the pericentriolar region of the cell and functions in recruiting cargo to centriolar satellites [57]. In our study, we found that wild-type endogenous BBS4 localizes to the pericentriolar region of HeLa cells as described (Figure 5). Similarly, the mutant BBS4 protein was also expressed and localized to the pericentriolar region of the cell, suggesting that the stable protein was produced and localized normally (Figure 5).

## Discussion

Though the eye phenotype of LCA and BBS has some overlap, they are still slightly different. Patients with LCA show sensory nystagmus, amaurotic pupils, and absent electrical signals on ERG. The BBS eye phenotype is called rod-cone dystrophy, which means visual acuity, dark adaptation, and peripheral visual fields are affected [58]. Patients with BBS have attenuated light- and dark-adapted ERGs [59].

*BBS4* comprises 16 exons, and at least 17 mutant alleles have been reported, including nine missense/nonsense changes [60-63], three splicing changes [61] and five in/dels (Figure 3A) [61-63]. BBS4 is a pericentriolar protein with a key role in recruiting cargo to centriolar satellites and is required for microtubule anchoring and cell cycle progression [57]. The mechanism by which this new allele causes a phenotype resembling LCA without other typical defects associated with BBS is likely due to a partial loss of function that mainly affects the retinal function of BBS4. Based on our cell culture assays, this mutation does not affect protein production or localization. Therefore, loss of function is likely due to either a retina-specific reduction of BBS4 protein levels or a specific functional defect within the retina. The latter case may be more likely since overexpression of the mutant *BBS4* allele does not rescue phenotypes in the zebrafish model. In mice, *BBS4* is known to play an important role in the proper transport of key phototransduction proteins from photoreceptor inner segments to outer segments [49]. The new allele we report here genetically separates *BBS4* function in the retina versus other tissues, and therefore provides a useful reagent to specifically interrogate *BBS4* function in retinal dystrophy.

In summary, we show that the combination of homozygosity mapping, whole-exome enrichment, and next-generation sequencing provides a sensitive, accurate, and cost-efficient tool for genetic analysis. Large numbers of variants, including rare variants, exist in each individual. As a result, although filtering against dbSNP and 1000 Genomes data can dramatically reduce the number of variants, many remain. Therefore, the main challenge of the whole exome sequencing approach is to devise a strategy to dramatically reduce the number of candidate variants for follow-up studies. One approach is to take advantage of genetic mapping information. In our case, homozygosity mapping narrowed the candidate locus to an 11.2 Mb region. Intersection of candidate variants and the candidate locus resulted in a single remaining variant. In addition, high-throughput functional assays can be used to further test candidate genes. The small size, high fecundity, and experimental tractability of zebrafish make it an excellent system for cost-effective and rapid screening of numerous candidates. Most human genes are conserved in zebrafish, which can therefore serve as a valid model for predicting gene function in humans. The combination of next-generation sequencing, genetic mapping, and high-throughput functional assays will greatly accelerate the identification of human disease genes.

## Appendix 1.

To access the data, click or select the words “Appendix 1.” This will initiate the download of a compressed (pdf) archive that contains the file.
